# Histological Study of Peanut Hull: Initial Barrier Against Fungal Invasion?

**DOI:** 10.3390/plants15121849

**Published:** 2026-06-15

**Authors:** Birat Sapkota, Nirmal Joshee

**Affiliations:** Agricultural Research Station, Fort Valley State University, 1005 State University Drive, Fort Valley, GA 31030, USA; birat.sapkota@fvsu.edu

**Keywords:** aflatoxin, hull, histochemistry, image analysis, peanuts

## Abstract

Research on the cataloging of microstructures and chemical compound localization in peanut hulls in relation to fungal tolerance remains limited. The hull (pericarp) is the first physical interface with the soil environment and may contribute to defense against fungal invasion. Here, hull microstructure and histochemical localization of alkaloid-like compounds, cellulose, lignin, starch, and total proteins were characterized across reproductive developmental stages R3–R6 in three commercially grown cultivars (Georgia-06G, Georgia-12Y, and Georgia-18RU). Stained sections were examined by light and fluorescence microscopy, and images were quantified in Fiji-ImageJ as stained area percentage. Among the compounds studied, the highest area percentages were observed at later stages (R5 and R6). Alkaloid-like compounds, cellulose, and starch were higher at the R5 stages of G-18 (9.61 ± 0.75), G-12Y (22.96 ± 5.84), and G-06 (6.31 ± 1.13) respectively, while lignin and total proteins were highest at the R6 stage of G-18 (respectively, 14.49 ± 1.43 and 13.90 ± 1.45). The lowest histochemical presence for most metabolites occurred in the early stages (R3–R4). This indicates that hull maturation is accompanied by increased physical (sclerenchyma and lignified cells) and biochemical (alkaloid-like compounds, proteins) features consistent with protective roles. As the analysis was based on representative sections and regions of interest (ROI)-level quantification, the results are intended to guide future studies on hull-mediated defense and breeding for *Aspergillus* tolerance.

## 1. Introduction

Peanuts (*Arachis hypogaea* L.; Fabaceae), are an important source of oil, folate, antioxidants, protein, and essential fatty acids [1]. The peanuts grown in the USA are of four types: Runner, Virginia, Spanish, and Valencia. Georgia-06G (G-06), Georgia-12Y (G-12Y), and Georgia-18RU (G-18) are some of the popular runner-type peanut cultivars grown in Georgia. World peanut production was more than 52 million metric tons in 2025, with the United States, at fourth place, producing more than three million metric tons [2]. Peanut production in the US has been facing a serious issue for decades, namely, aflatoxin contamination. Aflatoxin is a carcinogenic compound produced by *Aspergillus* spp., mainly *A. flavus* and *A. parasiticus*, during both the pre- and post-harvest stages. Although different strategies have been used in peanut production to reduce the *A. flavus* infection and subsequent aflatoxin biosynthesis, it remains a major issue, causing a yearly loss of around $126 million to the US peanut industry [3].

During pollination, the legume (fruit) first appears above ground but after fertilization, the developing pod grows down into the ground by the proliferation and elongation of a special structure called a ‘peg’, from cells below the ovary. In general, the pod is made up of two seeds, each with a papery seed coat [4]. The reproductive developmental stages in peanuts are classified as R1 (bloom), R2 (peg formation), R3 (pod formation), R4 (full length pod), R5 (seed formation), R6 (final seed size), R7 (mature) and R8 (mature for harvesting) [5]. A mature peanut seed can be divided into hull or pericarp, seed coat or testa, cotyledon, and embryo [6]. The peanut hull (pericarp) is the first structure formed after fertilization and its higher lignification protects the developing embryo from mechanical, biotic, and abiotic stresses [5] Peanut hulls are also an excellent source of cellulose and crude fiber and have high liquid absorbency, chemical inertness, and biodegradability [7,8]. Peanut hulls of various cultivars exhibit remarkable antioxidant potential, with high amounts of total polyphenol, flavonoid, and amino acid contents [9,10]. Studies have shown that peanut hulls have antioxidant and antimicrobial properties that provide an inhibitory effect against insect-pest attack [11,12]. Considering this, secondary metabolites, including terpenoids, alkaloids, flavonoids, and tannins, have been studied in medicinal plant extracts and their roles in biofilm inhibition, membrane disruption, and oxidative stress induction have been studied [13,14].

Plants are a source of many metabolites that vary in structure, quantity, location, and activity within the same species [15]. Besides direct toxic effects against phytopathogens, natural metabolites can promote root and shoot development and/or disease resistance by activating host systemic defenses [16]. In addition, secondary metabolites in plants are also identified as herbivore repellents; pollinator attractants; allelopathic agents; toxicity protection sources; UV-light shielding structures; and signal transduction providers [16]. The Fabaceae family is widely distributed worldwide and is recognized by its invasive ability and its high content of specialized metabolites [17].

Histochemical studies provide an approach to evaluate the presence of various primary and secondary metabolites in plant tissues and may help in the study of antimicrobial potential or determinations of possible other roles of compounds present. These studies, using specific stains, provide valuable information on the presence and extent of distribution in various types of cells and tissues. Histochemical studies on fresh tissue of *Solanum palinacanthum*, using Lugol stain to detect starch, and Coomassie blue for proteins, were conducted, revealing distinctive structural features and their ecological importance [18].

This research employed microscopic techniques to identify micromorphological structures present on the peanut hull and look into the possibility of their functional relationships in providing potential antifungal properties, full or partial. Histochemical studies were carried out, aiming to prepare paraffin sections and stain and quantify secondary metabolites among the peanut cultivars during reproductive developmental stages that may have anti-microbial or antifeedant properties, as suggested in various plant species. We wanted to explain how these metabolites are distributed in hull tissues and how they vary with maturation and cultivar. To our knowledge, no previous study has examined the histochemical localization of these compounds specifically in the peanut hull layers, and the present study addresses this gap. We hypothesized that later developmental stages (R5–R6) would demonstrate increased lignification and the accumulation of defense-related metabolites that may have roles as fungal deterrents, which is indicative of improved mechanical and biochemical barriers.

## 2. Materials and Methods

### 2.1. Classification of Peanuts According to Maturity Stage

**Figure 1:**
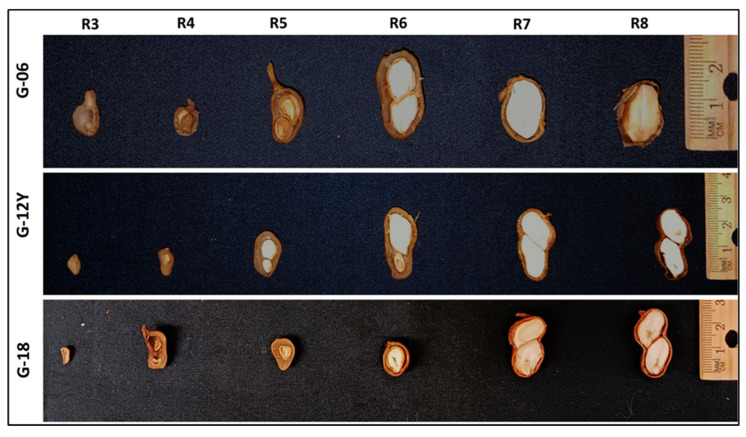
Reproductive development stages (R3–R8) of peanut cultivars (G-06, G-12Y, and G-18).

Sample collection: Seeds (Birdsong Peanuts, Blakely, GA, USA) of G-06, G-12Y, and G-18 (Table 1) were sown at the Fort Valley State University (FVSU) peanut field. The fruits/seed samples were then collected during the harvesting season (October to November 2022). Seeds of each cultivar were classified into six reproductive developmental stages, specifically, R3, R4, R5, R6, R7, and R8, according to their maturity (Figure 1) [5], and stored in 70% ethanol at 4 °C until further use.

### 2.2. Paraffin Sectioning and Light Microscopy

The peanut seeds at different developmental stages were fixed in formaldehyde 37%: glacial acetic acid: 95% ethanol: distilled water (FAA) in a ratio of 2:1:10:7 (*v*/*v*) and dehydrated through an ascending series of ethanol (30%, 50%, 70%, 90%, and 100%). The plant sample in 100% ethanol was kept at 65 °C and paraffin pellets (Type 9, Epredia, Portsmouth, NH, USA) were added for infiltration. The infiltration period was calibrated (two to four weeks) depending on the seed maturity. Then, the specimens were embedded in molten paraffin (58 °C), and 7–10 µM thick sections were cut using the rotary microtome (HM 355S Microm, Thermo Fisher Scientific, Waltham, MA, USA). Serial sections were cut and placed in the water bath at 43 °C, adding 10 mL/L Surgipath Sta-On (Leica Biosystems Richmond, Inc., Richmond, IL, USA). The sections were placed on microscopic slides (Globe Scientific Inc., Mahwah, NJ, USA), and the excess water was drained by placing slides on slide holders (Thermo Fisher Scientific, Waltham, MA, USA). Slides with sections were kept on a hot plate at 38–40 °C overnight to remove traces of water and allow the sections to stick properly. Deparaffinization was facilitated by xylene (at 37 °C) to remove paraffin and retain sections on the slide. The sections were used for both morphological and histochemical studies.

For the morphological studies, the sections were stained in aqueous (aq.) toluidine blue ‘O’ (0.1%). The stained sections were observed under a light microscope (Olympus BX43, LabX, Center Valley, PA, USA) and images were captured. Images of unstained section were used as control. The cellular localization of different compounds was captured using the Olympus Software under the microscope (Olympus BX43, LabX, Center Valley, PA, USA). White-balance calibration was not performed, which did not affect the qualitative localization findings, but this could be a standard procedure in future studies for better clarity between unstained and stained sections.

Toluidine Blue ‘O’ staining: After deparaffinization, the slides were submerged in toluidine Blue ‘O’ (0.1%) for 30 to 45 s. Stained sections on the slide were then placed in distilled water for 45 s to 1 min to remove non-specifically bound stain. The water was drained and slides were dried using lint-free wipes. Sections were mounted with acrytol (Leica Biosystems Richmond, Inc., Richmond, IL, USA), covered with a cover slip and observed.

### 2.3. Histochemical Localization of Primary and Secondary Metabolites

The staining protocols followed for histochemical localization of chemical compounds of interest are listed in Table 2. Only unstained controls were included in this study, to highlight the differences. A few modifications were made to optimize the protocol on which the experiments were based, and these are listed here:

i. Cellulose (Calcofluor-white)

The concentration of calcofluor solution used was 0.25% (*w*/*v*) and it was stained for 20 min.

ii. Lignin (Phloroglucinol–HCl)

A few drops of a saturated aqueous solution of phloroglucinol (10%) in 20% HCL were placed on the sections for one minute, and then the stain was drained and the remaining stains in the slides were wiped. The sections were mounted in same solution, covered with a cover slip and observed under light microscope.

iii. Total Proteins (Coomassie Brilliant Blue)

The concentration of Coomassie Brilliant Blue solution used was 0.25% (*w*/*v*) and the samples were stained for 15 min.

### 2.4. Quantification of Histochemical Staining

Digital images were analyzed using Fiji-ImageJ (ImageJ-win64) software [25]. For each stain, color thresholding was applied to distinguish positively stained tissue from background and unstained regions. The total hull area and the positively stained area within each region of interest (ROI) were measured, and staining was expressed as area%. Thus, for every cultivar × stage × metabolite combination, three ROIs (sub-samples) from representative sections were quantified (Table 3). Thresholds for each stain were manually determined based on the observation as to which value best identifies the maximum stained areas. The upper threshold was set at the value that captured the stained areas uniformly across all images within each metabolite class. We acknowledge that automated thresholding methods such as Otsu’s algorithm would improve objectivity, and these approaches are recommended for future studies.

### 2.5. Data Analysis

For each combination of cultivar, developmental stage, and metabolite, the mean and standard error (SE) of stained area% across the three ROIs were calculated and reported. These three ROIs represent technical sub-regions from representative sections of the same biological specimen and do not constitute independent biological replicates. Accordingly, no inferential statistical analyses were performed, and all comparisons between stages or cultivars should be interpreted as exploratory spatial and developmental patterns rather than statistically validated differences.

## 3. Results

### 3.1. Histological Study

**Figure 2:**
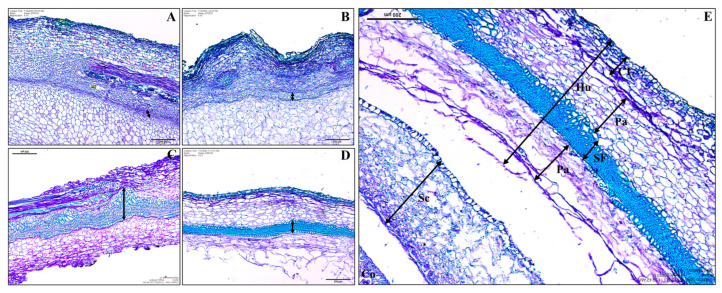
Sclerenchyma fibers in the hull at different developmental stages of G-12Y peanut cultivar (toluidine blue), 10×. (**A**) R3, (**B**) R4, (**C**) R5, (**D**) R6, and (**E**) different parts of peanut seed (G-12Y, R6). The double-arrowed lines (**A**–**D**) indicate the width of sclerenchyma fibers. (Hu = Hull, Sc = Seed coat, Co = Cotyledon, CL = Corky layer, Pa = Parenchyma cells, SF = Sclerenchyma fibers). The toluidine blue-stained image shown in Figure 2D is also used in Figure 3W to illustrate variation in the staining pattern of hull tissues among cultivars at different reproductive developmental stages. The scale bar indicates 200 μm.

**Figure 3:**
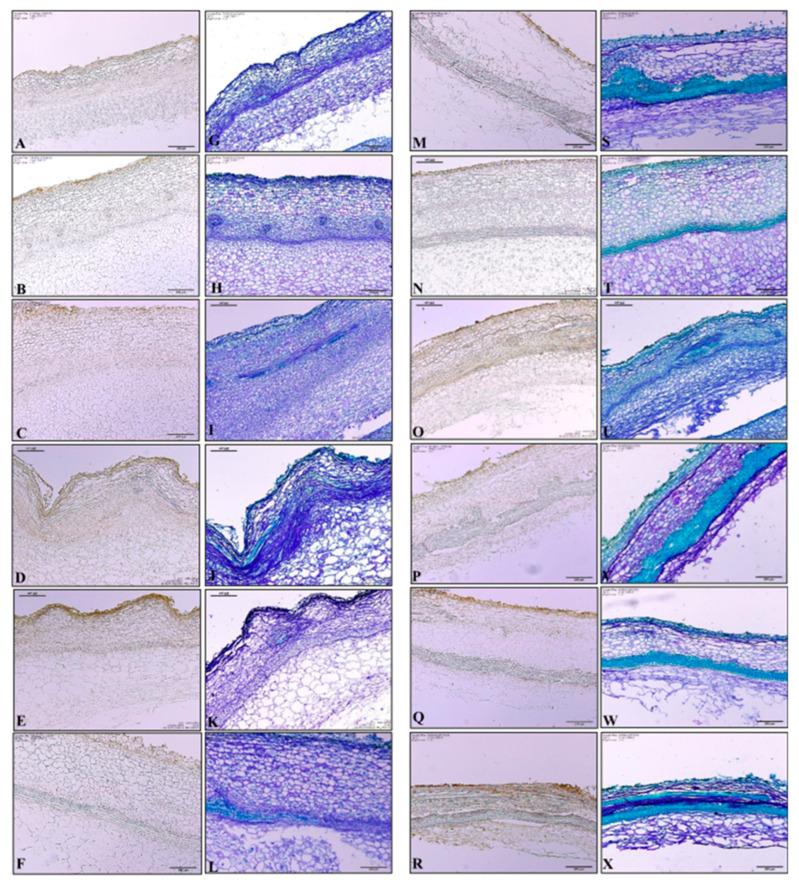
Toluidine blue staining in hull (10×). Control (Unstained) sections: R3: (**A**) G-06, (**B**) G-12Y, and (**C**) G-18; R4: (**D**) G-06, (**E**) G-12Y, and (**F**) G-18; R5: (**M**) G-06, (**N**) G-12Y, and (**O**) G-18; R6: (**P**) G-06, (**Q**) G-12Y, and (**R**) G-18. Stained: R3: (**G**) G-06, (**H**) G-12Y, and (**I**) G-18; R4: (**J**) G-06, (**K**) G-12Y, and (**L**) G-18; R5: (**S**) G-06, (**T**) G-12Y, and (**U**) G-18; R6: (**V**) G-06, (**W**) G-12Y, and (**X**) G-18. The scale bar indicates 200 μm. The same unstained hull sections were used as controls in the histological (Figure 3) and histochemical (Figure 4, Figure 5, Figure 6, Figure 7 and Figure 8) studies to illustrate how the staining pattern changes in the same anatomical region before and after application of each stain.

Toluidine blue was used for the morphological study of the hull that provided information regarding the changes in its cellular structure (Figure 2A–D). It helped to observe the distinct differences in different parts of a peanut seed (Figure 2E). As the seeds matured, the differences in the width or thickness of parenchyma cells and sclerenchyma fibers were evident in the hulls of all the cultivars (Figure 3).

### 3.2. Histochemical Localization

The presence of metabolites was studied with the use of specific stains, given their localization in different parts of the peanut seeds. Histochemical study provided information regarding the localization of chemical compounds of interest in different parts of the seeds of peanut cultivars at different reproductive developmental stages.

#### 3.2.1. Alkaloid-like Compounds

**Figure 4:**
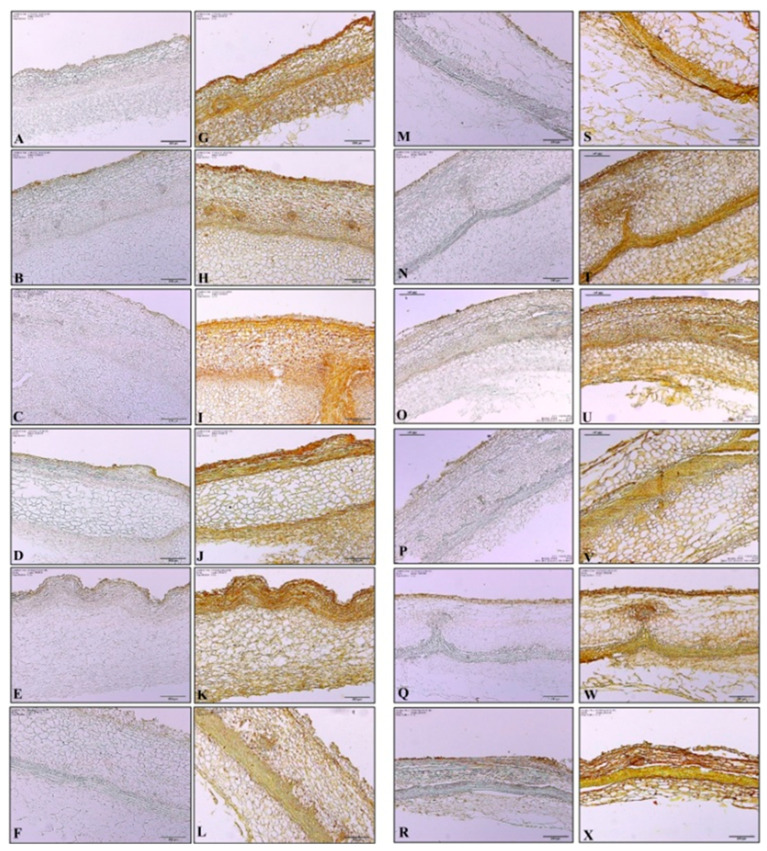
Dragendorff’s staining for the localization of alkaloid-like compounds in hull (10×). Control sections: R3: (**A**) G-06, (**B**) G-12Y, and (**C**) G-18; R4: (**D**) G-06, (**E**) G-12Y, and (**F**) G-18; R5: (**M**) G-06, (**N**) G-12Y, and (**O**) G-18; R6: (**P**) G-06, (**Q**) G-12Y, and (**R**) G-18. Stained: R3: (**G**) G-06, (**H**) G-12Y, and (**I**) G-18; R4: (**J**) G-06, (**K**) G-12Y, and (**L**) G-18; R5: (**S**) G-06, (**T**) G-12Y, and (**U**) G-18; R6: (**V**) G-06, (**W**) G-12Y, and (**X**) G-18. The scale bar indicates 200 µm.

The presence of alkaloid-like compounds in the hulls of peanut cultivars at different developmental stages was observed, and appeared brown when stained with Dragendorff’s reagent (Figure 4G–L,S–X), compared to unstained controls (Figure 4A–F,M–R). The presence of alkaloid-like compounds was observed almost in every part of the hull; however, darker stain was observed in the corky layer and sclerenchyma fibers at all reproductive developmental stages, suggesting the denser presence of alkaloid-like compounds (Figure 4).

#### 3.2.2. Cellulose

**Figure 5:**
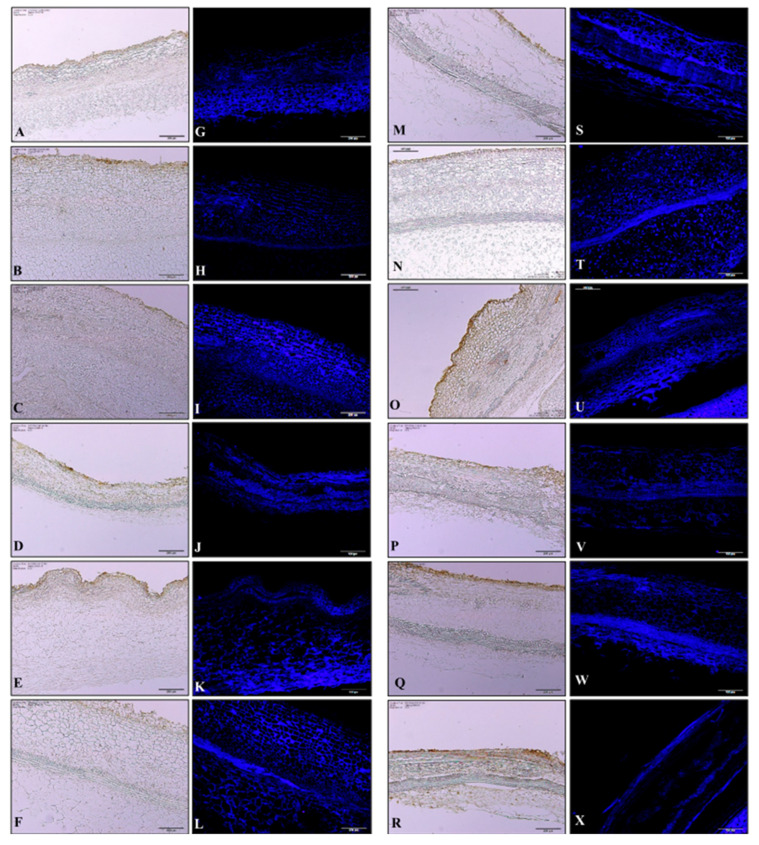
Calcofluor white staining (fluorescence) for the localization of cellulose in hull (10×) Control sections: R3: (**A**) G-06, (**B**) G-12Y, and (**C**) G-18; R4: (**D**) G-06, (**E**) G-12Y, and (**F**) G-18; R5: (**M**) G-06, (**N**) G-12Y, and (**O**) G-18; R6: (**P**) G-06, (**Q**) G-12Y, and (**R**) G-18. Stained: R3: (**G**) G-06, (**H**) G-12Y, and (**I**) G-18; R4: (**J**) G-06, (**K**) G-12Y, and (**L**) G-18; R5: (**S**) G-06, (**T**) G-12Y, and (**U**) G-18; R6: (**V**) G-06, (**W**) G-12Y, and (**X**) G-18. The scale bar indicates 200 µm.

The presence of cellulose in the hulls of peanut cultivars at different developmental stages was observed using a DAPI filter under UV light. The sections were stained in calcofluor white, and cellulose emitted blue fluorescence under UV light (Figure 5G–L,S–X) in comparison to the (control) unstained images (Figure 5A–F,M–R). The presence of cellulose was observed in different parts of the hull at all reproductive developmental stages of peanut cultivars (Figure 5).

#### 3.2.3. Lignin

**Figure 6:**
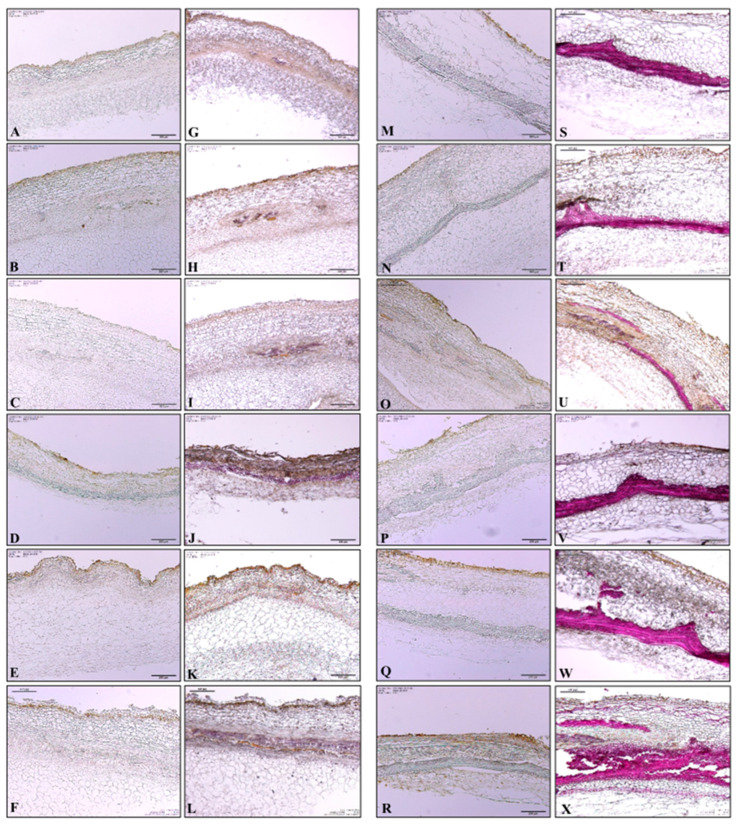
Phloroglucinol–HCl staining for localization of lignin in hull (10×). Control sections: R3: (**A**) G-06, (**B**) G-12Y, and (**C**) G-18; R4: (**D**) G-06, (**E**) G-12Y, and (**F**) G-18; R5: (**M**) G-06, (**N**) G-12Y, and (**O**) G-18; R6: (**P**) G-06, (**Q**) G-12Y, and (**R**) G-18. Stained: R3: (**G**) G-06, (**H**) G-12Y, and (**I**) G-18; R4: (**J**) G-06, (**K**) G-12Y, and (**L**) G-18; R5: (**S**) G-06, (**T**) G-12Y, and (**U**) G-18; R6: (**V**) G-06, (**W**) G-12Y, and (**X**) G-18. The scale bar indicates 200 µm.

The presence of lignin in the hulls of peanut cultivars at different developmental stages was observed, stained red using the Phloroglucinol–HCl stain (Figure 6G–L,S–X), in comparison to the control (unstained) images (Figure 6A–F, M–R). The lignification in the hull was observed more in later stages (R5 and R6) in comparison to the earlier stages (R3 and R4).

#### 3.2.4. Starch

**Figure 7:**
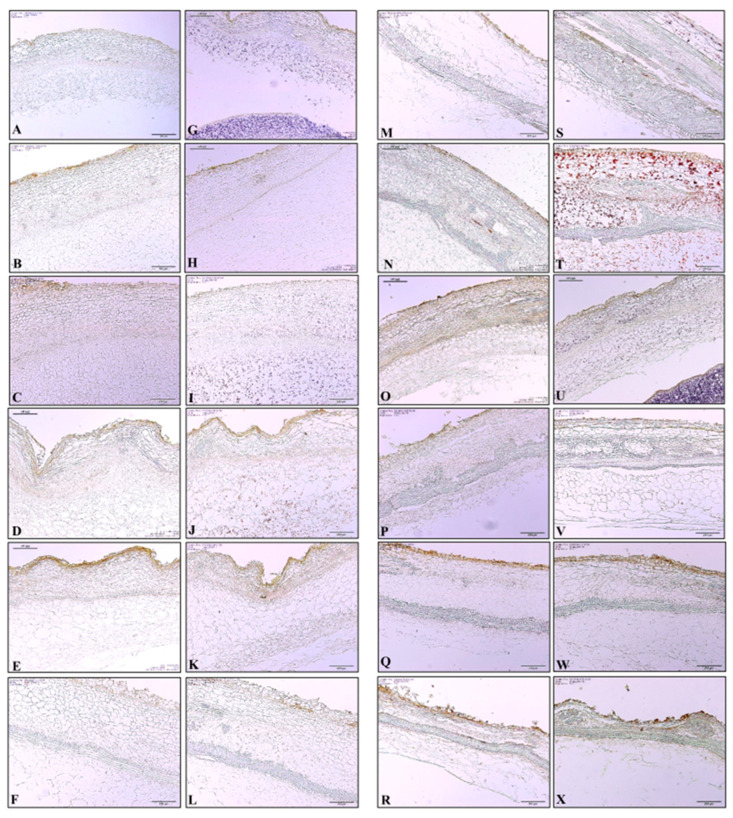
IKI staining for localization of starch in hull (10×). Control sections: R3: (**A**) G-06, (**B**) G-12Y, and (**C**) G-18; R4: (**D**) G-06, (**E**) G-12Y, and (**F**) G-18; R5: (**M**) G-06, (**N**) G-12Y, and (**O**) G-18; R6: (**P**) G-06, (**Q**) G-12Y, and (**R**) G-18. Stained: R3: (**G**) G-06, (**H**) G-12Y, and (**I**) G-18; R4: (**J**) G-06, (**K**) G-12Y, and (**L**) G-18; R5: (**S**) G-06, (**T**) G-12Y, and (**U**) G-18; R6: (**V**) G-06, (**W**) G-12Y, and (**X**) G-18. The scale bar indicates 200 µm.

The presence of starch in the hulls of peanut cultivars at different developmental stages was observed, stained purple to dark brown using Lugol’s reagent (Figure 7G–L,S–X), in comparison to the control (unstained) images (Figure 7A–F,M–R). The presence of starch was observed in the parenchyma cells of the hull mostly at the R3, R4, and R5 stages (Figure 7), while it was present at near-negligible levels in the R6 stage (Figure 7).

#### 3.2.5. Total Proteins

**Figure 8:**
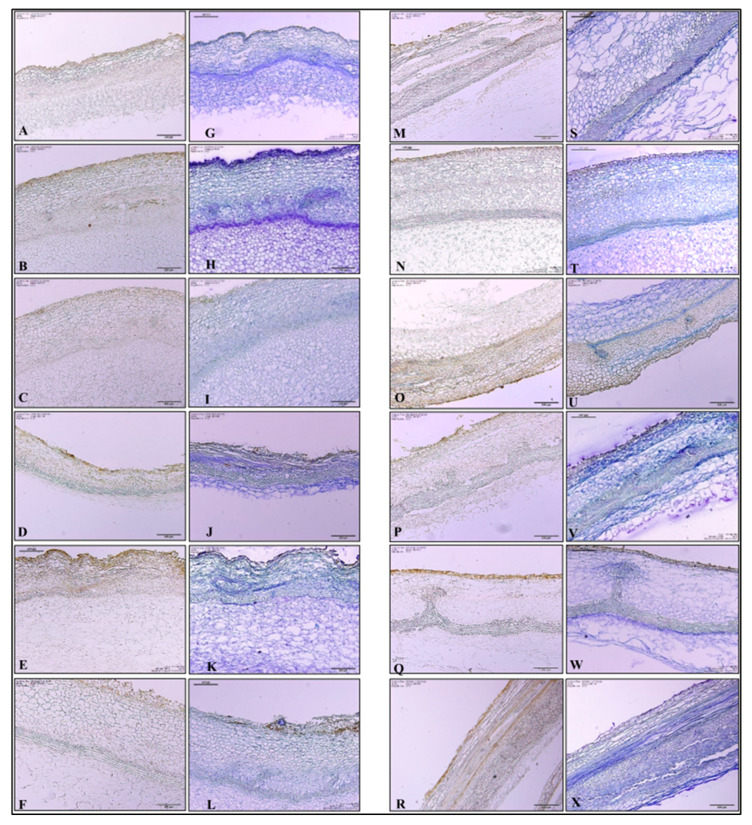
Coomassie Brilliant Blue staining for localization of total proteins in hull (10×). Control sections: R3: (**A**) G-06, (**B**) G-12Y, and (**C**) G-18; R4: (**D**) G-06, (**E**) G-12Y, and (**F**) G-18; R5: (**M**) G-06, (**N**) G-12Y, and (**O**) G-18; R6: (**P**) G-06, (**Q**) G-12Y, and (**R**) G-18. Stained: R3: (**G**) G-06, (**H**) G-12Y, and (**I**) G-18; R4: (**J**) G-06, (**K**) G-12Y, and (**L**) G-18; R5: (**S**) G-06, (**T**) G-12Y, and (**U**) G-18; R6: (**V**) G-06, (**W**) G-12Y, and (**X**) G-18. The scale bar indicates 200 µm.

The presence of total proteins in the hulls of peanut cultivars at different developmental stages was observed as stained blue using Coomassie Brilliant Blue (Figure 8G–L,S–X) in comparison to the control (unstained) images (Figure 8A–F,M–R). Total proteins were observed to be distributed in every part of the hull, but were found to be dense around the area of the corky layer and sclerenchyma fibers of the hull at all reproductive developmental stages of the peanut cultivars (Figure 8).

### 3.3. Quantification and Data Analysis

**Figure 9:**
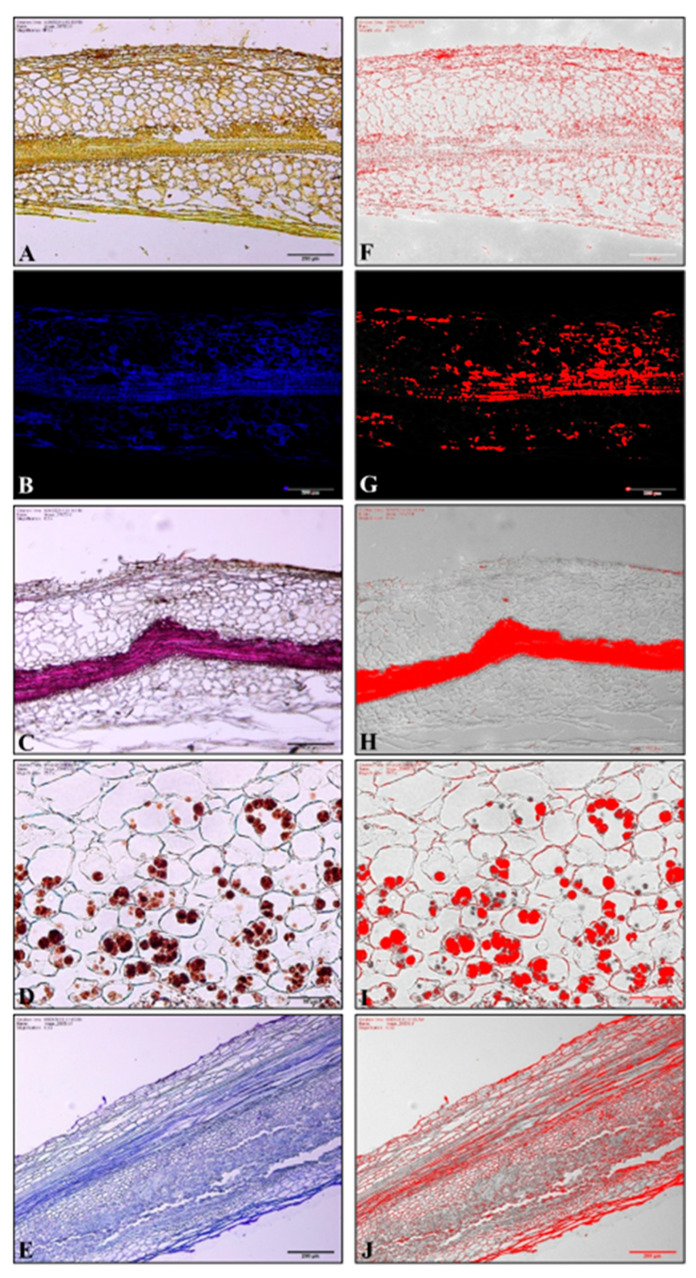
Sections before (stained sections) and after processing through Fiji-ImageJ for selected metabolites. Before processing through Fiji-ImageJ: (**A**) Alkaloid-like compounds, (**B**) Cellulose, (**C**) Lignin, (**D**) Starch, and (**E**) Total proteins. After processing through Fiji-ImageJ: (**F**) Alkaloid-like compounds, (**G**) Cellulose, (**H**) Lignin, (**I**) Starch, and (**J**) Total proteins.

The histochemical study was followed by image analysis that provided quantitative data as to the presence of metabolites in the hull (Figure 9). The comparative study of area% for the metabolites was done between the reproductive developmental stages among the cultivars (Table 4). The highest percentages of stained area for the alkaloid-like compounds, cellulose, lignin, starch and total proteins were calculated in G-18 at the R5 stage (9.61 ± 0.75), G-12Y at the R5 stage (22.96 ± 5.84), G-18 at the R6 stage (14.49 ± 1.43), G-06 at R5 (6.31 ± 1.13), and G-18 at the R6 stage (13.90 ± 1.45), respectively. Also, the lowest area% for alkaloid-like compounds, cellulose, lignin, starch, and total proteins were calculated in G-18 at the R3 stage (5.30 ± 1.97), G-12Y at the R3 stage (1.59 ± 0.62), G-06 at the R3 stage (0.09 ± 0.01), G-18 at the R4 stage (0.41 ± 0.04), and G-18 at the R3 stage (4.90 ± 1.19), respectively.

## 4. Discussion

Structural modifications and the presence of specific chemical compounds in plants play an important role in imparting tolerance against biotic and abiotic factors. The hull of the peanut (*Arachis hypogaea* L.) is the outermost structural layer of the fruit, positioned as the first biological interface between the developing seed and the soil environment. As such, it is the primary anatomical structure most likely to encounter *A. flavus* and *A. parasiticus* during pre- and post-harvest conditions [5]. In this study, histological and histochemical approaches were applied to characterize the microstructure and distribution of selected compounds across reproductive developmental stages R3–R6 in three commercially important Georgia cultivars. The results are discussed below with reference to the results obtained. Where peanut-specific literature on hull histochemistry could not be identified, this is explicitly noted, as the absence of such data itself reflects the novelty of the present investigation.

### 4.1. Hull Morphology and Sclerenchyma Development

Toluidine blue staining of paraffin sections revealed distinct tissue layers in the peanut hull, namely, an outer corky layer, a central zone of parenchyma cells, and an inner band of sclerenchyma fibers, with the seed coat and cotyledon visible beneath (Figure 2E). These observations are consistent with the general anatomical description of peanut pericarp reported by Pattee and Young [6] and with the reproductive development framework described by Mendu et al. [5]. A progressive increase in sclerenchyma fiber thickness from R3 to R6 was observed across all three cultivars (Figure 2A–D), indicating active secondary cell wall deposition as the hull matures. Mendu et al. [5] noted that higher lignification is associated with protection of the developing embryo from mechanical and biotic stresses, and the present histological results are consistent with that description. This developmental change is biologically significant because thicker, lignified sclerenchyma has been directly linked to reduced fungal penetration in plant tissues: in rice, OsMYB30-driven sclerenchyma reinforcement was shown to physically impede penetration by *Magnaporthe oryzae*, and activation of lignin biosynthesis-associated genes was shown to strengthen sclerenchyma cells against pathogen invasion [26,27]. While a direct parallel in peanut hull has not been established, the present histological data suggest a structurally analogous process may operate during peanut reproductive maturation. To our knowledge, no prior study has quantified stage-specific sclerenchyma development across R3–R6 in G-06, G-12Y, or G-18 cultivars, making the present observations a novel morphological baseline for these commercially grown Georgia cultivars.

### 4.2. Alkaloids-like Compounds

Dragendorff’s staining revealed alkaloid-like compounds distributed throughout the hull in all three cultivars across all developmental stages (R3–R6), with notably denser staining in the corky layer and sclerenchyma fibers (Figure 4; Table 4). Area% values for alkaloid-like staining were relatively consistent across developmental stages within each cultivar, ranging from 5.30 ± 1.97 (G-18, R3) to 9.61 ± 0.75 (G-18, R5), suggesting a constitutive rather than strongly stage-induced accumulation pattern. The dense localization of alkaloid-like compounds in the corky layer and sclerenchyma positions them at the precise interface most likely to be encountered by invading fungal hyphae, which is relevant to the central question of this study. Histochemical data specifically on alkaloid localization in *A. hypogaea* hull tissues could not be identified in the published literature, highlighting this as a novel observation. In other plant systems, alkaloids and alkaloid-containing fractions have demonstrated antifungal activity: different types of alkaloids have been found effective for the partial to complete inhibition of fungal spore germination [28], and the crude alkaloid extract from seeds of *Carthamus tinctorius* L. showed significant reduction in *Aspergillus* spp. spore germination, an effect attributed to membrane disruption and interference with metabolic activity [29]. Additionally, quinolizidine alkaloid-rich extracts from *Lupinus* spp. (Fabaceae) have demonstrated significant inhibition of mycelial growth of phytopathogenic fungi, including *Fusarium oxysporum*, *Sclerotium rolfsii*, and *Rhizoctonia solani*, under in vitro conditions [30,31]. These observations from related Fabaceae members and other alkaloid-containing seeds provide a plausible interpretive context for the dense alkaloid-like staining in the outer hull tissues of peanut. It should be noted that Dragendorff’s reagent, while widely used for alkaloid histochemistry [18,21], can react with some non-alkaloid nitrogen-containing compounds; therefore, the present results should be regarded as evidence of alkaloid-like compound localization rather than definitive identification of specific alkaloid molecules. Future work using HPLC-MS or similar analytical approaches would be needed to characterize the specific alkaloid composition of peanut hull tissues. We acknowledge that ethanol storage may result in partial extraction of low-molecular-weight metabolites such as alkaloids and phenolic compounds, which could be a limitation of this study that can be addressed in future work by processing fresh or immediately fixed tissue.

### 4.3. Cellulose

Calcofluor white staining under UV fluorescence microscopy revealed cellulose distributed in hull cell walls at all developmental stages and in all three cultivars (Figure 5; Table 4). Notably, G-12Y showed higher area% values at R4 and R5 (18.50 ± 4.68 and 22.96 ± 5.84, respectively) compared to G-06 and G-18 at the same stages, which may reflect cultivar-specific differences in cell wall architecture or in the timing of wall deposition and remodeling. By R6, cellulose area% values generally declined, particularly in G-06 (3.96 ± 0.93), which may be consistent with compositional shifts during hull maturation. Histochemical data on cellulose distribution specifically in peanut hull layers across R3–R6 were not identified in the published literature, underscoring the novelty of these observations. Cellulose is one of the major components of cell walls and plays a role as a barrier to fungal invasion [32]; disruption of the cell wall by biotic or abiotic factors is known to trigger plant defense mechanisms [32], and decreased cellulose biosynthesis has been shown to alter cell wall integrity, activating signaling pathways related to plant defense [33]. In barley, decreased cellulose content in epidermal cell walls was associated with compromised resistance to powdery mildew [34], while other studies have shown that impaired cellulose synthesis results in dwarfing but increased disease resistance [35,36]. The cultivar-level differences in cellulose area% observed here, particularly the higher values in G-12Y, deserve further investigation in the context of field-measured susceptibility data for these cultivars. It is important to acknowledge that cellulose does not act in isolation; the mechanical contribution of cellulose to wall strength depends on its interaction with hemicelluloses and pectins, factors which were not analyzed in the present study and which should be incorporated in future investigations for a more complete model of hull wall composition.

### 4.4. Lignin

Phloroglucinol–HCl staining revealed that lignin showed the clearest and most consistent developmental increase across all cultivars and stages. Area% values were very low at R3 and R4 (G-06: 0.09 ± 0.01 at R3, 0.11 ± 0.01 at R4; G-18: 0.15 ± 0.02 at R3, 0.19 ± 0.05 at R4) but increased dramatically at R5 and R6 (G-06: 11.68 ± 1.28 at R5, 10.37 ± 0.11 at R6; G-18: 1.37 ± 0.62 at R5, 14.49 ± 1.43 at R6; Table 4). This pattern is temporally consistent with the histologically observed thickening of sclerenchyma-associated tissues between early and later developmental stages (Figure 2A–D and Figure 6) and aligns with the description by Mendu et al. [5] that peanut hull lignification increases during reproductive maturation to protect the developing embryo. In other plant systems, enhanced lignification has been directly linked to resistance against fungal pathogens: enhanced lignin content in rice conferred broad-spectrum resistance to blast disease and bacterial leaf blight caused by *Magnaporthe oryzae* and *Xanthomonas oryzae*, respectively [37]; plants exposed to pathogen infection or deficient in cellulose biosynthesis showed higher lignification that increased mechanical strength and improved tolerance to cell wall-degrading enzymes released by pathogens [38,39]; and activation of lignin biosynthesis-associated genes resulted in accumulation of lignin subunits in sclerenchyma cells that resisted *M. oryzae* penetration in rice [27]. Conversely, downregulation of lignin in *Medicago sativa* L. increased alfalfa resistance against *Colletotrichum trifolii* by activating defense responses and upregulating pathogenesis-related genes [40], illustrating that the relationship between lignification and defense is context-dependent and may vary by pathosystem. The R5–R6 stages at which dramatic lignin increases were observed in this study correspond to critical pre-harvest windows of *A. flavus* infection risk, making the timing of this developmental lignification particularly relevant to aflatoxin contamination biology. However, as no direct fungal assays were conducted in this study, the connection between hull lignin levels and *Aspergillus* resistance in peanuts remains a working hypothesis requiring experimental validation.

### 4.5. Starch

IKI (Lugol’s) staining revealed starch present primarily in parenchyma cells of the hull at R3, R4, and R5, with markedly reduced or absent signal at R6 (Figure 7; Table 4). The highest starch area% values were observed in G-06 at R5 (6.31 ± 1.13) and G-12Y at R5 (5.50 ± 2.71), while values at R6 were low across all cultivars (G-06: 0.90 ± 0.24; G-12Y: 1.82 ± 0.52; G-18: 1.46 ± 0.14). This stage-dependent decline is biologically consistent with the known role of pericarp parenchyma as a transient carbon reserve during early pod development, with starch being mobilized as the seed approaches final size [41]. To our knowledge, no published study has specifically characterized Lugol’s staining patterns in peanut hull layers across these developmental stages for these cultivars. Previous studies in starch-stress relations have shown that plants remobilize their starch reserves to release energy, sugars, and derived metabolites to help mitigate stress [41]; degradation of starch in response to abiotic stress has been correlated with improved tolerance in various plants, including freezing tolerance in *Physcomitrella patens* [42], and greater starch degradation was found in drought-resistant varieties of broad bean compared to drought-sensitive varieties [43]. In the context of biotic stress, a recent study in maize showed that upon *A. flavus* invasion, genes involved in carbohydrate synthesis were significantly downregulated, while genes involved in energy synthesis were significantly upregulated, indicating that maize converted its stored carbohydrate into energy to fight the invasion [44]. The depletion of parenchymal starch in peanut hull by R6 may therefore reflect an energetic remodeling consistent with a shift toward mature tissue maintenance and defense readiness, though this interpretation remains speculative without direct metabolic or molecular data from this system.

### 4.6. Total Proteins

Coomassie Brilliant Blue staining revealed that total protein was broadly distributed across hull tissues at all developmental stages, with consistently denser staining concentrated in the corky layer and sclerenchyma fibers compared to parenchyma cells in all three cultivars (Figure 8; Table 4). G-18 at R6 showed the highest total protein area% (13.90 ± 1.45), and G-06 at R3 was also notably high (11.74 ± 0.78). Plants have developed a defense mechanism against biotic stress that includes a series of complex molecular mechanisms, including pathogenesis-related (PR) proteins, which are among the most well-characterized defense proteins [45,46,47]. Proteins are actively involved in enzymatic reactions, performing regulatory roles in various cellular processes that contribute to the maintenance of cell structure as well as defense against pathogens [48], and various membrane-bound proteins such as histidine kinase DesK and Hik33 identify the effect of abiotic stresses and trigger the plant defense mechanism [49]. While the Coomassie assay detects total proteins and does not distinguish specific protein classes, the persistent concentration of protein signal in the outer, mechanically reinforced hull tissues is compatible with the localization of defense-relevant protein pools in these regions. Importantly, a study in various peanut genotypes found that total proteins were higher in insect-resistant genotypes compared to others [50], making it the peanut-specific study closest linking total protein levels to pest resistance. This precedent is consistent with the interpretation that the protein-rich outer hull tissues observed here may have a functional contribution to resistance; however, further work using immunohistochemistry or proteomics would be needed to characterize the specific proteins present. A recent study specifically characterizing peanut seed coat secondary metabolites also identified protease inhibitors and defense-related proteins as components of the seed coat barrier against *Aspergillus* infection [51], providing additional support for the hypothesis that protein-based defense features are present in peanut outer tissues.

### 4.7. Integrated Interpretation and Implications for Hull-Mediated Defense Against Aspergillus

Across all five compounds analyzed, the present dataset reveals a coherent pattern: peanut hull maturation from R3 to R6 is accompanied by coordinated anatomical and histochemical changes that collectively support a model of progressive barrier reinforcement. Lignification increases most dramatically at R5–R6, coinciding with sclerenchyma fiber thickening; alkaloid-like compounds and proteins are concentrated in the corky layer and sclerenchyma throughout development; cellulose is distributed in cell walls at all stages with cultivar-level variation; and starch transitions from a parenchymal reserve at early stages to a depleted state at R6.

These findings are directly relevant to *A. flavus* biology in peanut: the R5–R6 stages correspond to pre-harvest pod maturation windows when soil moisture stress conditions are most conducive to *A. flavus* colonization and aflatoxin biosynthesis [5]. The hull’s progressive structural and chemical reinforcement during this window, particularly the lignin surge and the persistent alkaloid-like and protein staining in outer tissues, is consistent with a model in which the mature hull presents both a physical impediment to fungal hyphal penetration and a localized chemical environment that may be unfavorable to fungal growth. Peanut hull has been documented to possess antioxidant and antimicrobial properties [9,11,12], and peanut seed coat secondary metabolites have recently been shown to contribute to biochemical barriers against *Aspergillus* infection [51]. Furthermore, comparative metabolomics of peanut hull in the context of *Aspergillus* resistance has identified secondary cell wall components as important factors in distinguishing resistant and susceptible genotypes [52], supporting the relevance of the structural features characterized in this study.

As this is the first detailed histological and histochemical localization study in peanuts, it has a few limitations. The use of three technical ROI sub-samples rather than independently grown biological replicates; the absence of formal positive staining controls; potential partial extraction of low-molecular-weight metabolites during ethanol storage; and the non-inclusion of other relevant cell wall components (hemicelluloses, pectins, cutin, and suberin). Future studies should address these limitations by incorporating biological replication (3–5 plants per cultivar × stage) with two-way ANOVA statistical analysis; conducting *A. flavus* spore germination inhibition assays with stage-specific hull extracts; applying higher-magnification imaging and immunohistochemistry for specific defense proteins; and expanding the histochemical panel to include pectins, hemicelluloses, and suberin.

## 5. Conclusions

In this study, histological, histochemical, and image-analysis approaches were applied to characterize the microstructure and distribution of selected compounds in the hulls of three commercially important Georgia peanut cultivars (G-06, G-12Y, and G-18) across reproductive developmental stages R3–R6. To our knowledge, this constitutes the first systematic, stage-resolved histochemical characterization of alkaloid-like compounds, cellulose, lignin, starch, and total proteins specifically in the hull layers of these cultivars, addressing a gap in the published literature, given that most *A. hypogaea* research has focused on bulk chemical extraction or molecular profiling of seed tissues rather than tissue-level localization in the hull.

Across all cultivars, hull maturation was accompanied by clear developmental changes in tissue morphology, including progressive thickening of sclerenchyma fibers, and in the spatial distribution of the compounds analyzed. Most notably, lignin area% values increased dramatically from R3–R4 to R5–R6, concurrent with sclerenchyma reinforcement, indicating active secondary cell wall deposition during hull maturation. Alkaloid-like compounds and total proteins were detected at all stages, with denser localization in the mechanically reinforced corky layer and sclerenchyma fibers, indicating the preferential concentration of these compounds in the outermost hull tissues throughout development. Starch was present in parenchyma cells primarily during the early stages and was markedly reduced by R6, which is consistent with carbon remobilization during late-stage seed development.

As several of these compounds have been associated with antifungal or defense-related functions in prior research on peanut and related plant species, their presence and developmental accumulation patterns are consistent with—but do not by themselves confirm—a role for the peanut hull as a protective barrier against *Aspergillus* invasion. The present study was intentionally scoped as a descriptive baseline characterization; the barrier hypothesis remains a working proposition that requires direct experimental validation.

We propose the following concrete next steps: (1) testing peanut hull extracts from each cultivar at R5 and R6 stages against *A. flavus* and *A. parasiticus* in spore germination bioassays; (2) conducting properly replicated histochemical studies with 3–5 independent plants per cultivar × stage, enabling statistical comparison by two-way ANOVA; (3) characterizing the specific alkaloid and protein composition of hull tissues using HPLC-MS and proteomics approaches; and (4) expanding the histochemical panel to include suberin, pectins, and hemicelluloses. These investigations will bridge the anatomical and histochemical baseline established here with functional and molecular mechanisms, ultimately supporting the development of *Aspergillus*-tolerant peanut cultivars through evidence-based breeding strategies.

## Figures and Tables

**Table 1:** Descriptions of different peanut cultivars growing in Georgia [19].

S.N.	Georgia-06G (G-06)	Georgia-12Y (G-12Y)	Georgia-18RU (G-18)
1.	A high yielding, runner-type cultivar with large sized seed; released in 2006.	A high yielding, medium-late maturing, runner-type cultivar with a medium sized seed; released in 2012.	A high-yielding runner-type variety; released in 2018.
2.	A high level of Tomato Spotted Wilt Virus (TSWV) resistance.	TSWV- and white-mold-resistant and susceptible to *Rhizoctonia* Limb Rot.	It is resistant to TSWV and leaf-scorch [20].
3.	Good yield potential in a wide range of conditions.	Due to later maturity, Georgia-12Y is less suitable for later planting dates (after May 15).	The ideal planting window is between late April and late May, regarding yield potential.

**Table 2:** Staining protocols for metabolites studied.

S.N.	Metabolites	Stain Used	Procedure	Color of Metabolites	Reference
1	Alkaloids	Dragendorff’s reagent solution	Sections stained for 15 min.	Orange–red to brown	[18,21]
2	Cellulose	Calcofluor Method (Fluorescence)	Calcofluor solution (0.25%, *w*/*v*) for 20 min	Dark blue/black	[22,23]
3	Lignin	Phloroglucinol–HCl Test	A large drop of a saturated aqueous solution (phloroglucinol (10%) in 20% HCl) placed on the slide.	Red violet/pink	[22,23]
4	Starch	Lugol’s reagent	Sections submerged in Lugol’s reagent for 10 min	Dark blue to black	[22,23]
5	Total Proteins	Coomassie Brilliant Blue	Sections stained in Coomassie blue solution (0.25%, *w*/*v*) for 15 min.	Blue color	[18,24]

**Table 3:** Steps followed for the image analysis of pictures acquired by light and fluorescence microscopy, using Fiji-ImageJ software.

Steps/Parameter	Light Microscopy	Fluorescence Microscopy
1. Open Image	Open the captured brightfield image.	Open the captured fluorescence image.
2. Set Scale	Zoom into scale → draw straight line → *Analyze* → *Set Scale* → enter known distance and µm units → apply.	Same procedure: Zoom → draw line on scale → *Analyze* → *Set Scale*.
3. * Background Processing	For alkaloid-like compounds and lignin: Process → Subtract Background (100 px, Light Background, Create Background, Sliding Paraboloid, Disable smoothing).	Not used.
4. Image Type Conversion	Convert to RGB stack, then select the green channel.	Convert image to 8-bit.
5. Thresholding	Press Shift + T → set metabolite-specific thresholds (minimum–maximum): Alkaloid-like compounds: 0–145 Lignin: 0–100 Starch: 0–60 Total proteins: 0–95	Press Shift + T → apply threshold (minimum–maximum) 25–75.
6. * Tissue Cleanup/Segmentation	Remove unwanted tissues (hull, seed coat, cotyledon) via polygon selection → Edit → Cut. Applies to all metabolites except lignin and starch.	Same tissue removal procedure using the polygon tool.
7. Area Measurement	Press M or use Analyze → Measure to extract area values.	Press M or use Analyze → Measure.
8. Saving Output	*File* → *Save As*	*File* → *Save As*
Notes	* Additional background subtraction step used only for alkaloid-like compounds and lignin. (For lignin: Stained area % = Total stained area % − Seed coat and cotyledons area %)	Used primarily for cellulose images captured under fluorescence microscopy.

**Table 4:** Area (%) of metabolites in peanut hull (Mean ± S.E.).

Cultivars	Reproductive Developmental Stages	Alkaloid-like Compounds	Cellulose	Lignin	Starch	Total Proteins
G-06	R3	7.45 ± 0.53	13.46 ± 2.09	0.09 ± 0.01	1.06 ± 0.11	11.74 ± 0.78
	R4	6.20 ± 0.88	16.11 ± 7.42	0.11 ± 0.01	1.62 ± 0.61	10.67 ± 0.89
	R5	7.67 ± 0.80	12.71 ± 2.98	11.68 ± 1.28	6.31 ± 1.13	9.56 ± 0.25
	R6	8.98 ± 0.76	3.96 ± 0.93	10.37 ± 0.11	0.90 ± 0.24	10.29 ± 0.34
G-12Y	R3	6.76 ± 0.90	1.59 ± 0.62	0.12 ± 0.01	0.44 ± 0.12	11.70 ± 2.01
	R4	9.18 ± 1.06	18.50 ± 4.68	2.00 ± 0.00	0.75 ± 0.15	6.43 ± 1.07
	R5	8.32 ± 1.84	22.96 ± 5.84	1.94 ± 1.62	5.50 ± 2.71	8.68 ± 0.55
	R6	7.50 ± 1.35	12.90 ± 1.49	10.14 ± 1.60	1.82 ± 0.52	5.74 ± 0.14
G-18	R3	5.30 ± 1.97	16.49 ± 8.21	0.15 ± 0.02	1.06 ± 0.68	4.90 ± 1.19
	R4	6.88 ± 0.89	15.33 ± 2.74	0.19 ± 0.05	0.41 ± 0.04	7.19 ± 1.22
	R5	9.61 ± 0.75	7.36 ± 4.58	1.37 ± 0.62	1.62 ± 0.49	6.23 ± 0.66
	R6	8.59 ± 0.60	8.74 ± 0.45	14.49 ± 1.43	1.46 ± 0.14	13.90 ± 1.45

## Data Availability

The raw data supporting the conclusions of this article will be made available by the authors on request.
